# Combined Transcriptomic and Proteomic Analysis of Perk Toxicity Pathways

**DOI:** 10.3390/ijms22094598

**Published:** 2021-04-27

**Authors:** Rebeka Popovic, Ivana Celardo, Yizhou Yu, Ana C. Costa, Samantha H. Y. Loh, L. Miguel Martins

**Affiliations:** MRC Toxicology Unit, University of Cambridge, Gleeson Building, Tennis Court Road, Cambridge CB2 1QR, UK; rp636@cam.ac.uk (R.P.); ivana.celardo@uniroma1.it (I.C.); yzy21@cam.ac.uk (Y.Y.); anacarinaecosta@gmail.com (A.C.C.); shyl2@cam.ac.uk (S.H.Y.L.)

**Keywords:** *Drosophila*, *Drosophila* protein kinase RNA (PKR)-like ER kinase (dPerk), ER stress, unfolded protein response, activating transcription factor 4 (ATF4)

## Abstract

In *Drosophila*, endoplasmic reticulum (ER) stress activates the protein kinase R-like endoplasmic reticulum kinase (dPerk). dPerk can also be activated by defective mitochondria in fly models of Parkinson’s disease caused by mutations in *pink1* or *parkin*. The Perk branch of the unfolded protein response (UPR) has emerged as a major toxic process in neurodegenerative disorders causing a chronic reduction in vital proteins and neuronal death. In this study, we combined microarray analysis and quantitative proteomics analysis in adult flies overexpressing dPerk to investigate the relationship between the transcriptional and translational response to dPerk activation. We identified *tribbles* and *Heat shock protein 22* as two novel *Drosophila* activating transcription factor 4 (dAtf4) regulated transcripts. Using a combined bioinformatics tool kit, we demonstrated that the activation of dPerk leads to translational repression of mitochondrial proteins associated with glutathione and nucleotide metabolism, calcium signalling and iron-sulphur cluster biosynthesis. Further efforts to enhance these translationally repressed dPerk targets might offer protection against Perk toxicity.

## 1. Introduction

As we age, neurodegenerative diseases are becoming a prominent health problem worldwide. Despite large-scale efforts to treat these diseases, current therapies only offer symptomatic relief without the possibility of curative treatments. The unique genetic, pathological and clinical signatures of individual neurodegenerative diseases have focused scientific research on the disease-specific protein-centric mechanism of pathology. However, it is becoming increasingly clear that neurodegenerative diseases share a common subcellular signature. At present, the unfolded protein response (UPR) is perceived to be a generic feature of most neurodegenerative diseases (reviewed in [1]). Modulation of the UPR therefore represents a promising target for therapies designed to delay or prevent neurodegeneration (reviewed in [2]).

The endoplasmic reticulum (ER) represents a dynamic network of interconnected sheets and tube-like structures [3] in eukaryotic cells. The primary function of the ER is to act as a factory for proteins, coordinating the synthesis, folding, maturation and post-translational modifications of proteins. Certain pathological conditions, such as neurodegeneration, are characterised by an overproduction of unfolded or misfolded proteins leading to ER stress. To combat this problem, the ER activates an evolutionarily conserved adaptive response known as the UPR [4], which acts to restrict the protein folding load via translation repression [5] in concert with the transcriptional activation of specific genes encoding ER-resident chaperones and the ER-associated degradation machinery (reviewed in [6]).

The UPR is transduced via three protein kinases, namely, protein kinase RNA (PKR)-like ER kinase (PERK), inositol-requiring enzyme 1 (IRE1) and activating transcription factor 6 (ATF6), activated by the presence of unfolded proteins in the ER. While ATF6 and IRE1 primarily act via induction of a transcriptional programme [7,8,9], PERK activation leads to an overall translational shutdown, mediated by the phosphorylation of the eukaryotic translation initiator factor-2 α (eIF2α) [5]. The eIF2 complex is responsible for the methionyl-tRNA delivery, which is necessary for the initiation of protein synthesis by ribosomes. Phosphorylated eIF2α (phospho-eIF2α) can competitively bind eIF2, thereby inhibiting the GTP hydrolysis required for the initiator methionyl tRNA to base pair with the initiation codon. Consequently, the cap-dependent translation is inhibited at the initiation step due to the failure of the 80S ribosome complex to assemble (reviewed in [10]).

During ER stress, reduced protein synthesis serves to conserve energy and resources and thus enable cells to reconfigure gene expression to alleviate stress damage. However, the synthesis of some molecules is required to cope with the perturbed proteostasis, as their protein products are necessary for the resolution of ER stress. Thus, phospho-eIF2α also leads to the preferential translation of specific transcripts that facilitate adaptation to a specific stress condition, suggesting that the UPR requires translational reprogramming via alternative mechanisms of translation initiation (reviewed in [11]). In mammalian cells, phospho-eIF2α is required for the regulated expression of several proteins, such as activating transcription factor 4 (ATF4) and C/EBP homologous protein (CHOP) as well as mRNAs encoding heat shock and UPR proteins, such as ER chaperone binding immunoglobulin protein (BiP) and eIF2α phosphatase growth arrest and DNA damage-inducible protein (GADD34) [12,13,14,15]. While these alternative mechanisms of translation have not been fully elucidated to date, studies suggest that 5′ untranslated region (UTR) cis-acting sequences, such as upstream open reading frames (uORFs) and internal ribosome entry sites (IRES), confer the ‘privileged’ translation of these mRNAs in spite of eIF2α inhibition (reviewed in [16]).

Mutations in *PINK1* or *PARKIN*, two genes in mitochondrial quality control cause neurodegeneration in some autosomal recessive forms of Parkinson’s (PD) disease (reviewed in [17]). In *Drosophila melanogaster*, mutations in either *pink1* or *parkin* cause mitochondrial dysfunction and the degeneration of dopaminergic neurons [18].

We have recently shown that in *Drosophila pink1* or *parkin* mutants, defective mitochondria also give rise to ER stress signalling by activating the Perk branch of the UPR. Enhanced ER stress signalling in *pink1* and *parkin* mutants is mediated by contacts between defective mitochondria and the ER [19] and causes the upregulation of transcripts coding for serine hydroxymethyl transferase (Shmt2) and NAD-dependent methylenetetrahydrofolate dehydrogenase (Nmdmc) in both cultured human cells and flies. *Shmt2* and *Nmdmc* are targets of dAtf4 involved in mitochondrial one-carbon metabolism [20]. When overexpressed in *pink1* or *parkin* mutants, either Shmt2 or Nmdmc is neuroprotective [20].

These observations indicate that first, transcripts belonging to one-carbon metabolism that are targets of Perk signalling are upregulated in fly models of neurodegeneration, and second, these transcripts need to be overexpressed to confer protection. Both observations lead to the hypothesis that *Drosophila* Perk (dPerk) target genes that are upregulated at the transcriptional level but fail to be translated are involved in neuroprotective pathways. To investigate this possibility, we interrogated the full cohort of transcripts and proteins altered by dPerk expression with the aim of identifying the full cohort of genes that might be upregulated at the transcript level but not at the protein level. Such a cohort can comprise potential protective pathways such as the mitochondrial one-carbon pathway.

Therefore, we combined microarray analysis of transcripts and tandem mass tagging (TMT) proteomic analysis, in adult flies overexpressing dPerk. We set to functionally characterised upregulated transcripts and their corresponding changes in proteins via pathway enrichment using a network analysis approach and upstream analysis (Cytoscape with ClueGO, STRING and iRegulon). In this study, we show that dPerk upregulation promotes the translation of a limited set of mRNAs, that function to resolve stress; these mRNAs include transcripts from the mitochondrial chaperone *Heat shock protein 22* (*Hsp22)*. Furthermore, we have identified a subpopulation of translationally repressed mitochondrial proteins that are suggested to be involved in nucleotide metabolism, calcium signalling and sulphur compound metabolic processes. Our study provides an insight into the dynamic nature of Perk signalling and identifies potential neuroprotective targets, whose roles in ER stress merit further research.

## 2. Results

### 2.1. Drosophila tribbles Is Regulated by dPerk and dAtf4

**Figure 1:**
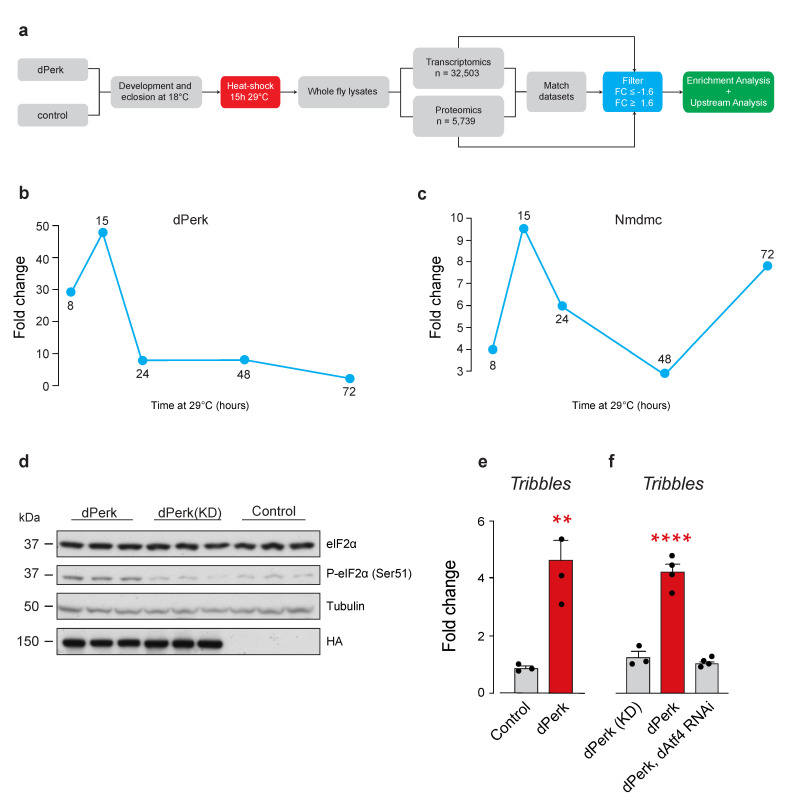
*Drosophila* dPerk regulates *tribbles* mRNA levels. (**a**) Workflow used for the characterisation of dPerk-dependent transcripts and proteins. To identify differentially expressed targets, transcripts and proteins were filtered by adjusted false discovery rate (FDR) and fold change (FC) values of 0.05 and ±1.6, respectively. The expression levels of transcripts and proteins were compared and further submitted for enrichment and upstream analysis. (**b**,**c**) *dPerk* or *Nmdmc* mRNA induction were assessed for different durations of the heat shock treatment (in hours (h)). The figure shows fold-change mRNA levels compared to the control, measured by real-time qPCR. (**d**) Western blot analysis of total and phospho-eIF2α protein levels in dPerk-HA, kinase-dead dPerk (KD)-HA and control flies following 15 h heat-shock at 29 °C. Whole-fly lysates were analysed using the indicated antibodies. (**e**) *Tribbles* (*trbl*), an endoplasmic reticulum stress marker, was upregulated in dPerk flies following 15 h of heat shock, as measured by real-time qPCR (mean ± SEM; asterisks, unpaired *t*-test). (**f**) *Trbl* overexpression is dAtf4 dependent and requires dPerk kinase function (15 h heat shock at 29 °C), as measured by real-time qPCR (mean ± SEM; asterisks, one-way ANOVA with Tukey’s multiple comparison test). Genotypes: Control: *tub*Gal80; *da*Gal4 > +, dPerk: t*ub*Gal80; *da*Gal4 > *dPerk*-HA, dPerk(KD): t*ub*Gal80; *da*Gal4 > *dPerk*-K671R-HA, dPerk, dAtf4 RNAi: *tub*Gal80; *da*Gal4 > d*Perk*-HA, *dAtf4* RNAi.

To study the cohort of transcriptional and protein changes in adult flies expressing *dPerk* we utilised the GAL4-UAS gene expression system adapted with a temperature-sensitive GAL80 protein (GAL80^ts^), expressed ubiquitously from the *tubulin 1α* promoter [21]. At the permissive temperature of 18 °C, ubiquitously expressed GAL80 protein is functional, and acts as a repressor of the GAL4 transcriptional activator by binding to the GAL4 protein. At restrictive temperatures above 29 °C, GAL80 can no longer bind GAL4 and its repressive function is therefore lost, thereby enabling the expression of the gene target placed under the control of the upstream activation sequence (UAS). Therefore, we generated a UAS fly line expressing an HA-tagged version of *dPerk*. Flies were raised at 18 °C and heat-shocked for 15 h at 29 °C following eclosion, enabling temporally controlled ubiquitous (*da*GAL4) expression of *dPerk* in adult flies, and they were processed for transcriptomics and proteomics analysis (Figure 1a). We chose to heat-shock the flies for 15 h, as this timepoint provided the highest level of detected *dPerk* expression by assessment of its mRNA levels (Figure 1b) and corresponded to a peak of expression of *Nmdmc*, a dAtf4 target (Figure 1c) [20].

We next confirmed the kinase activity of dPerk by examining the levels of its downstream target eIF2α. We compared the phosphorylation levels of eIF2α by detecting phospho-eIF2α by Western blotting. The overexpression of dPerk caused an increase in phospho-eIF2α levels, when compared to controls expressing either a kinase dead version of dPerk (K671R) or driver alone following a 15 h heat-shock (Figure 1d).

We have previously characterised a subnetwork of transcriptional changes present in *pink1* and *parkin* mutant flies, associated with the activation of the dPerk signalling pathway [20]. In this analysis we found in silico evidence for the induction of the tribbles pseudokinase 3 (TRB3), a negative regulator of ATF4-dependent transcription [22]. As we detected an upregulation of *Drosophila tribbles* (*trbl*) in our transcriptomics analysis (Supplementary Table S1), we next utilised quantitative real-time PCR (qRT-PCR) analysis and confirmed that *dPerk* expression caused an upregulation of *trbl* (Figure 1e). In mammalian cells, TRB3 is an ER stress marker and a target of ATF4 [23,24]. Therefore, we next tested the effects of downregulating *dAtf4* in flies expressing *dPerk*. We observed that downregulation of *dAtf4* blocked the increase in the mRNA levels of *trbl* caused by *dPerk* expression (Figure 1f). We conclude that *trbl* is a downstream target of dAtf4 regulated by dPerk in adult flies.

### 2.2. Divergence between the Upstream Transcriptional Regulators of dPerk-Induced Alterations in Transcripts and Proteins

Next, to assess the global cohort of dPerk-dependent alterations in mRNA and protein levels in adult flies we analysed the transcriptomic and proteomic dataset obtained from flies expressing *dPerk* using the workflow shown in Figure 1a. Microarray analysis, identified 32,500 distinct mRNA molecules (transcripts) that matched a total of 15,635 genes (Supplementary Table S1). We identified differentially expressed transcripts based on the predetermined thresholds set at ±1.6 fold-change for the FC value and 0.05 for the false discovery rate (FDR) value. This approach identified a total of 977 upregulated and 1022 downregulated transcripts, matching to 517 and 642 genes, respectively (Supplementary Tables S2 and S3). dPerk activation results in global translational repression, modulating protein synthesis under conditions of ER stress [25]. To determine how dPerk altered protein levels in flies we used quantitative proteomic analysis using the TMT technique.

Flies subjected to TMT proteomics were kept in conditions identical to those used for transcriptomics. Quantitative proteomic analysis identified 5795 proteins (Supplementary Table S1). We applied the same cut-off values as in the transcriptomic dataset (±1.6 fold-change for the FC value and 0.05 for the FDR) which yielded a list of 100 upregulated and 145 downregulated proteins (Supplementary Tables S4 and S5).

**Figure 2:**
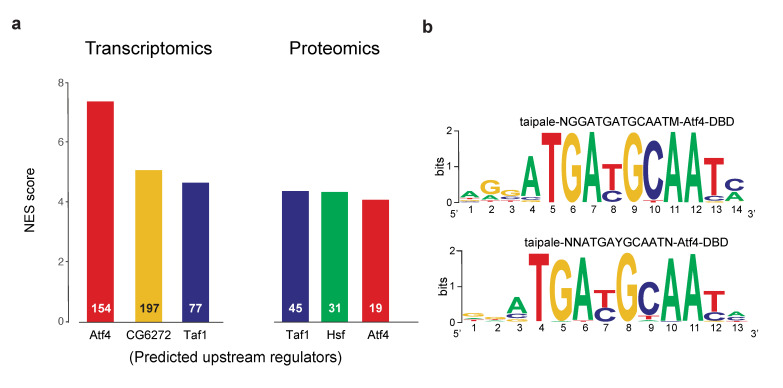
Upstream analysis of dPerk-upregulated transcripts and proteins. (**a**) Predicted upstream regulators of upregulated transcripts (left) and proteins (right) and their respective NES scores. The number of targets regulated by the specific transcription factor (TF) is presented on the bars. (**b**) Highest-ranking motifs of the ATF4 TF observed in upregulated transcripts. The analysis was performed using iRegulon, a Cytoscape application.

To understand the main transcriptional drivers of dPerk-mediated upregulation in transcripts and proteins we next used iRegulon, a computational method designed to reverse engineer the transcriptional regulatory network underlying coexpressed genes [26]. By subjecting the cohort of upregulated transcripts to iRegulon analysis we identified ATF4 as the primary transcriptional driver of the upregulation of mRNAs in dPerk expressing flies (Figure 2a,b, Supplementary Table S6). However, when an identical analysis was performed on the cohort of upregulated proteins, ATF4 failed to achieve the top score (Figure 2a, Supplementary Table S7). We conclude that, as predicted, ATF4 is the primary driver of dPerk-induced transcriptional changes in adult flies. However, the protein increases observed in these flies are induced by more complex regulatory mechanisms.

### 2.3. Pathway Analysis of dPerk-Dependent Alterations in Transcripts and Proteins

We subsequently compared the differentially expressed transcripts and proteins independently. We performed pathway enrichment analysis (PEA) using ClueGo, a Cytoscape plug-in that integrates Gene Ontology (GO) terms as well as KEGG and Reactome pathways creating functionally organised GO/pathway term networks [27]. This analysis involved separate right-sided hypergeometric tests, assessing GO term and pathway enrichment, for the upregulated and downregulated transcripts or proteins.

**Figure 3:**
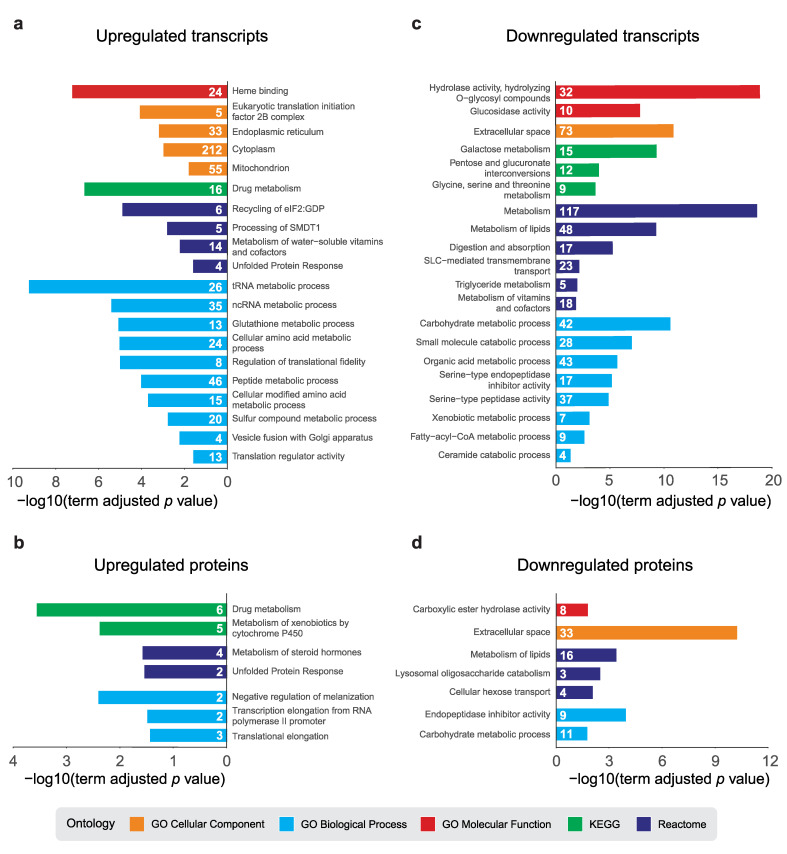
dPerk-dependent global expression changes. ClueGO pathway enrichment analysis (PEA) of differentially regulated molecules. Selected results from the Gene Ontology (GO) Cellular Component, GO Biological Process, GO Molecular Function, KEGG and Reactome, ontology terms and their respective −log10 (adjusted term *p*-value) are shown. The number of associated molecules of a specific functional term is displayed on the bars. ClueGO PEA of upregulated transcripts (**a**) and proteins (**b**). ClueGO PEA of downregulated transcripts (**c**) and proteins (**d**).

*dPerk* overexpression caused a transcriptional enrichment in UPR-related terms such as unfolded protein response, endoplasmic reticulum and recycling of eIF2:GDP that consist of canonical ER stress proteins such as Ire1, dPerk and eIF-2α (Figure 3a, Supplementary Tables S2 and S8). We also detected an enrichment of different aminoacyl-tRNA synthetases and terms closely linked to translation, such as tRNA, cellular amino acid and peptide metabolic processes. The terms related to drug and glutathione metabolism as well as haem binding were also significantly enriched, comprising glutathione S-transferases (GSTs), UDP-glycosyltransferases (UGTs), as well as Cytochrome P450 enzymes (CYPs). We also observed an upregulation of more than 50 mitochondrial transcripts (Figure 3a).

Functional analysis of upregulated proteins (Figure 3b, Supplementary Tables S4 and S9) indicated enrichment of two UPR proteins, dPerk and Ire1. We also observed upregulation of proteins that modulate transcriptional and translational elongation. In keeping with the results of the transcriptomic analysis, we observed enrichment in proteins involved in detoxification processes (i.e., drug metabolism, metabolism of xenobiotics by cytochrome p450 and metabolism of steroid hormones). We also observed enrichment of members of the serpin family that act as inhibitors of proteases required for the melanisation reaction, a *Drosophila* specific immune response [28,29].

Both downregulated transcripts (Figure 3c, Supplementary Tables S3 and S10) and proteins (Figure 3d, Supplementary Tables S5 and S11) were enriched in terms related to metabolism, specifically metabolism of carbohydrates and lipids. This result indicates that dPerk activation promotes the inhibition of several metabolic pathways.

### 2.4. Analysis of the dPerk-Induced Correlations between Transcript and Protein Levels

**Figure 4:**
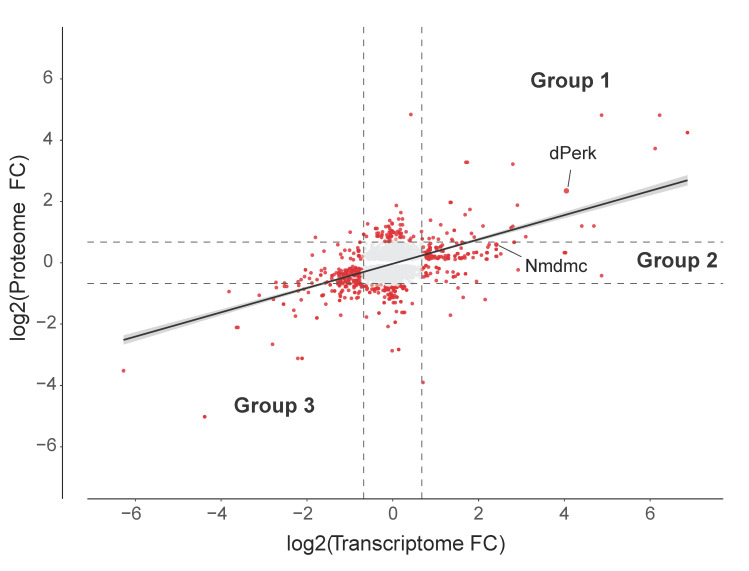
dPerk differential expression analysis (DEA). Comparison of dPerk-dependent fold change (FC) expression in the transcriptome and proteome (logged). Differentially expressed molecules are shown in red (adjusted false discovery rate (FDR) ≤ 0.05 and FC±1.6). The DEA shows a proportion of genes consistently regulated between the transcriptome and proteome (Pearson’s correlation, *r* = 0.5, *p*-value < 2.2 × 10^−16^) and a number of differentially regulated transcripts and proteins. The two subgroups of genes with upregulated transcripts, representing potentially protective pathways are indicated. Group 1 represents dPerk-dependent targets that show upregulation in both transcripts and proteins; group 2 represents translationally repressed targets that only show an upregulation of transcripts, with the absence of a change in protein levels. Group 3 represents targets that are repressed at both the transcript and protein level. dPerk and Nmdmc are indicated by black lines.

The UPR leads to an induction of a transcriptional programme that does not lead to a corresponding increase in translation, as transcripts are unable to produce their protein products due to a stall in cap-dependent translation. For example, we observed induction of the dAtf4 target gene *Nmdmc,* which was proposed to be neuroprotective in *pink1* or *parkin* mutants but observed that it was necessary to overexpress this gene to achieve neuroprotection [20]. This finding suggests that an increase in mRNA levels does not necessarily reflect a protein upregulation. To assess the relationship between the induction of mRNA and protein levels we compared the subgroups of transcripts matched to proteins (Figure 4, Supplementary Table S12). We defined two subgroups within the upregulated transcripts, first, genes that were upregulated at both the transcript and protein levels (group 1, Supplementary Table S13), and second, genes that were upregulated at the transcript level but not at protein level (group 2, Supplementary Table S14), as well as a subgroup of downregulated transcripts and proteins (group 3, Supplementary Table S15).

Next, we analysed the subgroups of upregulated transcripts. Our hypothesis is that genes belonging to group 2, consist of failed protective mechanisms. These factors, such as *Nmdmc,* might represent target pathways to achieve cellular protection from ER toxicity. The comparison between group 1 and group 2 genes revealed that *Nmdmc* transcript is in fact upregulated by dPerk, but overexpression of this kinase failed to induce an increase in Nmdmc protein levels (Figure 4).

We first set to examine group 1 targets. We established 27 targets that demonstrated upregulation in both transcripts and proteins (Table 1, Supplementary Table S13). Our results show that the expression levels of dPerk are upregulated after a 15 h heat-shock, thus validating our model. Resident ER proteins included Ire1, Asparagine synthetase (AsnS) and Sil1 nucleotide exchange factor (Sil1). Both *Ire1* and *AsnS* have previously been reported to be upregulated in response to ER stress [30,31]. Sil1, a GrpE-like protein, has been shown to function as a nucleotide exchange factor for an ER luminal chaperone BiP [32], and is required for protein translocation and folding in the ER [33].

Our analysis further identified two GTPases, Dgp-1 and CG2017, homologous to GTP-binding-protein 1 (GTPBP1) and GTPBP2, that are predicted to be involved in translational elongation. In *Drosophila*, Dgp-1 appears to play a role in the cellular defence against oxidative stress and has been found to be upregulated in *parkin* mutants [34], as well as in flies challenged with an inducer of oxidative stress, paraquat [35] and hyperoxic conditions [36].

Three mitochondrial proteins were also present, a mitochondrial chaperone Hsp22, Phosphoenolpyruvate carboxykinase 2 (Pepck2) and an uncharacterised protein CG34423. CG34423 is a suggested orthologue of the ATP synthase inhibitory factor subunit 1 (ATP5IF, 66% similarity) and has been found to be upregulated by Hsp22, suggesting that these two proteins might be functionally related [37].

**Figure 5:**
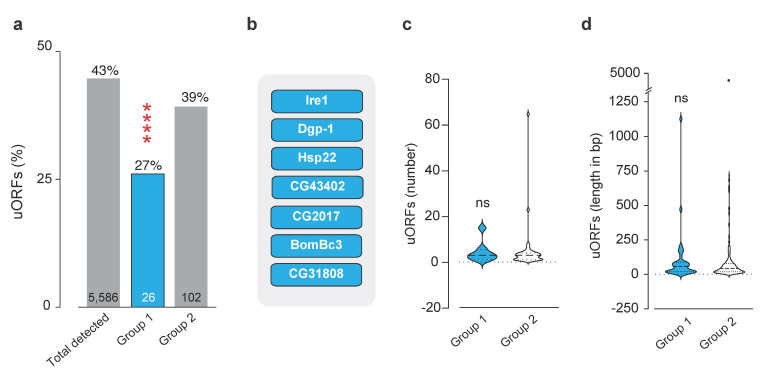
Analysis of upstream open reading frames (uORFs) in group 1 and group 2 genes. (**a**) Levels of uORFs, present either in the total number of molecules detected at both transcript and protein levels (total detected, Supplementary Table S16), compared to number of uORFs in group 1 or 2 (Supplementary Tables S17 and S18). Significance was calculated using a Monte Carlo test, see methods, *p* < 0.00001 and *p* = 0.10, respectively. The numbers inside the bars correspond to the total number of genes in each group. (**b**) Group 1 targets with uORFs. (**c**,**d**) uORF features were assessed, for respectively, (**c**), the number of uORFs per gene (median + 95% CI, Wilcoxon Rank Sum Test, *p* = 0.7) or (**d**), the uORF length per gene (median + 95% CI, Wilcoxon Rank Sum Test, *p* = 0.7).

The phosphorylation of eIF2α leads to the preferential translation of specific transcripts via alternative mechanisms of translation initiation such as uORFs and IRES (reviewed in [16]). We next investigated the degree of uORFs present in dPerk targets, detected at both transcript and protein level. We found that 43% of genes (2425 out of 5586) have uORFs (Figure 5a, Supplementary Table S16), consistent with what was previously reported [38]. Furthermore, 7 out of 26 group 1 targets (dPerk was excluded) (Figure 5b), and 40 out of 102 group 2 targets contain at least one uORF (Supplementary Tables S17 and S18).

Using a Monte Carlo-based approach, we determined that the number of genes in group 1 is a true positive whereas the number of genes from group 2 does not significantly differ from random sampling from the total population.

The impact that the uORFs can have on translation depends on many features, including their abundance or length (reviewed in [39]). However, upon inspection of these features we found no difference between the two groups (Figure 5c,d). We further investigated presence of IRES by examining the IRES database [40], but did not find any *Drosophila* targets with an IRES site within group 1.

### 2.5. Small Mitochondrial Chaperone Hsp22 Is a Novel dPerk/dAtf4 Signalling Target

Studies have shown that ER stress is able to modulate mitochondrial proteostasis and dynamics [37], as well as mitochondrial bioenergetics [41], in order to confer cellular adaptation to stress. Mitochondria and the ER communicate through mitochondrial ER contacts (MERCs), structural microdomains of parts of ER membranes in close apposition to mitochondrial outer membrane domains to regulate cell metabolism (reviewed in [42]). PERK has been identified as a component of MERCs [43]. In addition, ATF4 has been found to modulate mitochondrial stress responses [23]. All of these findings suggest that ER stress can modulate mitochondrial function.

Our analysis of group 1 members identified the mitochondrial Hsp22 to be upregulated at both transcript and protein levels. Hsp22 overexpression has been shown to increase *Drosophila* lifespan and resistance to oxidative stress, and its role in ageing suggests that Hsp22 helps to maintain mitochondrial integrity and function [44,45]. Hsp22 overexpressing flies display upregulation of genes related to mitochondrial energy production and protein biosynthesis, including genes of the mitochondrial oxidative phosphorylation system (OXPHOS) [46].

**Figure 6:**
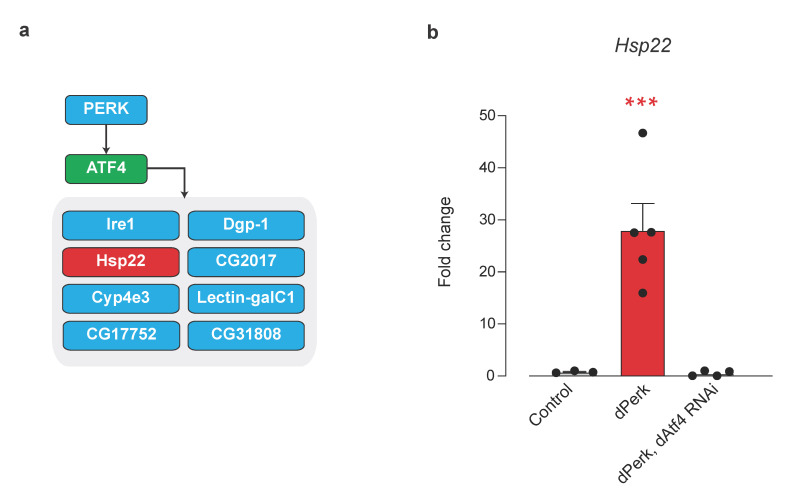
Mitochondrial chaperone Hsp22 is a target of dPerk/Atf4 signalling. (**a**) iRegulon analysis of group 1 genes suggests ATF4 (green) as a transcriptional regulator of *Hsp22* (red). Blue squares denote group 1 genes characterised by an upstream ATF4 binding motif. (**b**) *Hsp22* mRNA induction is regulated by dPerk and dAtf4. Expression levels were measured by real-time qPCR (mean ± SEM; asterisks, one-way ANOVA with Tukey’s multiple comparison test). Genotypes: Control: *tub*Gal80; *da*Gal4 > +, dPerk: *tub*Gal80; *da*Gal4 > *dPerk*-HA, dPerk, dAtf4 RNAi: t*ub*Gal80; *da*Gal4 > *dPerk*-HA, *dAtf4* RNAi. Adult flies were heat-shock for 15 h at 29 °C.

Analysis of *Hsp22* upstream regulators by iRegulon identified ATF4 as a potential transcriptional regulator of this mitochondrial chaperone (Figure 6a).

Therefore, we subsequently confirmed that *dPerk* expression caused an upregulation of *Hsp22* by qRT-PCR analysis (Figure 6b). We also found that downregulation of *dAtf4* blocked the increase in the mRNA levels of *Hsp22* caused by *dPerk* expression (Figure 6b). We conclude that *Hsp22* is a downstream target of dAtf4 regulated by dPerk in adult flies. We propose that Hsp22 may function to modulate mitochondrial function in response to ER stress.

**Figure 7:**
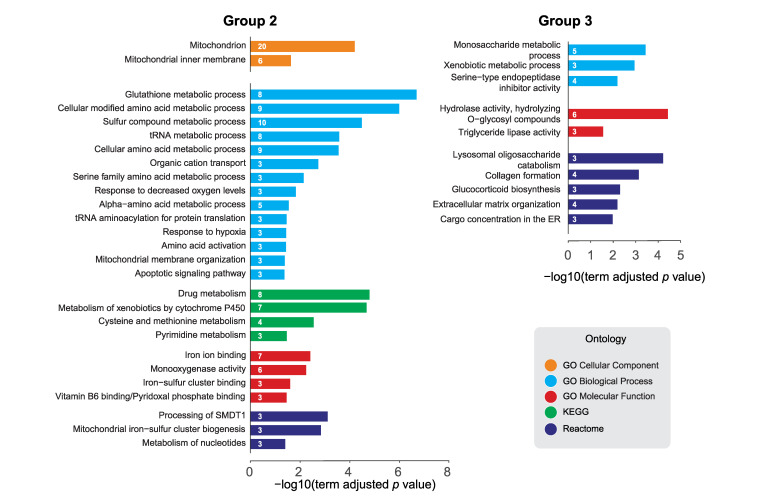
ClueGO pathway enrichment analysis (PEA) of dPerk-dependent targets. Genes that were upregulated at the transcript level but not at the protein level (group 2, **left**) and genes that were downregulated at the transcript and protein level (group 3, **right**) were analysed. PEA was performed using ClueGO, a Cytoscape application. Selected results from the Gene Ontology (GO) Cellular Component, GO Biological Process, GO Molecular Function, KEGG and Reactome, ontology terms and their -log10 (adjusted term *p*-value) are shown. The number of targets of a specific functional term is displayed on the bars.

We next examined group 2, defined by an upregulation in transcript levels without a corresponding change in protein levels. Group 2 consisted of 102 molecules (Figure 7, left, Supplementary Table S14). The ClueGo GO Cellular Component PEA right-sided hypergeometric test identified a strong enrichment in mitochondrial components, made up of 20 different mitochondrial targets including *Nmdmc* (Supplementary Table S19).

In addition to the enriched mitochondrial terms shown in Figure 7 (left), we observed enrichment in terms related to the metabolism of nucleotides as well as glutathione, drug and xenobiotic metabolism (Supplementary Table S20).

Finally, we looked at the subset of genes with downregulated transcript and proteins (group 3). This included 75 transcripts corresponding to 51 proteins (Supplementary Table S15). The ClueGo PEA right-sided hypergeometric test showed an enrichment in terms related to carbohydrate and triglyceride metabolism (Figure 7, right, and Supplementary Table S21), as observed in Figure 3c,d. Terms hydrolysing O-glycosyl compounds, lysosomal oligosaccharide catabolism and monosaccharide metabolic process display the highest significance, consisting of predominantly lysosomal alpha-mannosidases and maltase enzymes, which catalyze the catabolism of different carbohydrates or carbohydrates of glycoproteins [47,48].

### 2.6. Networks of Translationally Repressed Signalling Pathways Caused by dPerk Expression

**Figure 8:**
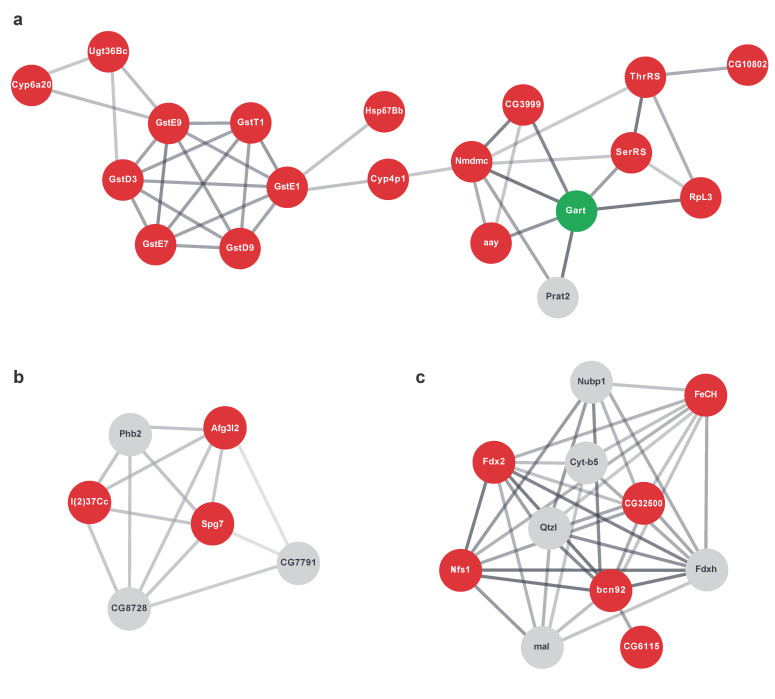
String network analysis of dPerk-dependent targets differentially regulated by the transcriptome and proteome. Genes upregulated at the transcript level but not at the protein level were analysed (group 2, red). Clusters were determined using the inbuilt Cytoscape stringApp Markov Cluster (MCL) algorithm. Clusters were enriched with first neighbours from the background transcriptomic network in order to find additional functionally related targets not recognised by the TMT labelling. The nodes representing molecules with an upregulation in transcript levels but no available protein value (grey) and molecules with an upregulation in transcripts and downregulation in protein levels (green) were added. Molecular pathways involved in glutathione and nucleotide metabolism (**a**), processing of essential mitochondrial calcium uniporter regulator (EMRE) (**b**) and iron-sulphur metabolism (**c**) are shown.

To define functional networks within group 2 and visualise the connectivity between molecules (nodes) we next used the STRING database. STRING collects, scores and integrates publicly available sources of protein-protein interaction information, complimenting them with computational predictions to provide comprehensive functional networks [49]. To reduce the complexity of our network we used the Markov cluster algorithm (MCL), a clustering algorithm for graphs. MCL clustering is based on the random walk principle and is used for the large-scale detection of protein clustering [50]. Using MCL we deconstructed the group 2 protein network until we defined functional cliques, clusters in which every node was connected to every other node in that cluster (Figure 8, red circles). Whereas protein molecules provide more information regarding the functionality inside the cell, the techniques currently used for proteome analysis are not at the scale of the methods used for the transcriptome, which are highly suitable for large-scale analyses. To circumvent this problem, we reconstructed our networks by reintroducing the predefined cliques into the background transcriptomics network and investigating the clique first neighbours to enrich for biological information lost by missing proteomics data (Figure 8, grey and green circles). After controlling for MCL cluster pathway specificity with stringApp functional enrichment analysis and through a literature review, we established three subnetworks within group 2 (Figure 8).

**Figure 9:**
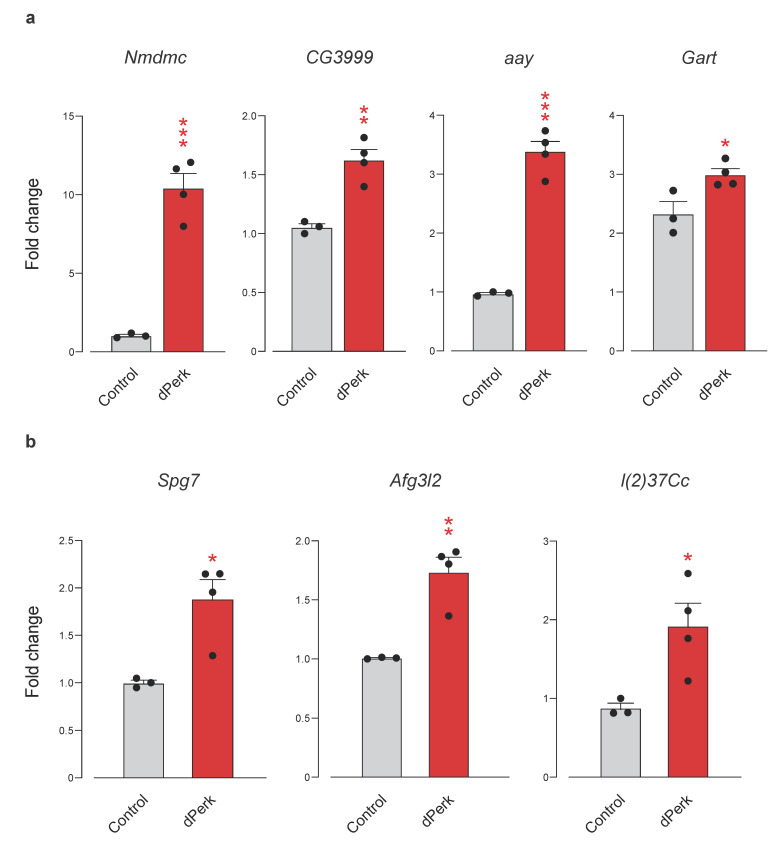
*dPerk* overexpression causes an upregulation in mRNA levels of molecules regulating nucleotide metabolism and mitochondrial calcium signalling. (**a**) *dPerk* overexpression results in transcriptional upregulation of nucleotide metabolism genes *NAD-dependent methylenetetrahydrofolate dehydrogenase* (*Nmdmc*), *CG3999*, a glycine dehydrogenase (GLDC) orthologue, *astray (aay*) and *GART trifunctional enzyme* (*Gart*). (**b**) *dPerk* overexpression results in transcriptional upregulation of the *Paraplegin* (*Spg7*), *AFG3 like matrix AAA peptidase subunit 2* (*Afg3l2*) and *lethal (2) 37Cc* (*l(2)37Cc*) genes involved in essential mitochondrial calcium uniporter regulator (EMRE) processing. Expression levels were measured by real-time qPCR. All bar plots show the mean ± SEM; asterisks, unpaired *t*-test. Genotypes, Control: *tub*Gal80; *da*Gal4 > +, dPerk: *tub*Gal80; *da*Gal4 > *dPerk*-HA. Adult flies were heat-shock for 15 h at 29 °C.

MCL clustering identified a large network consisting of two smaller highly connected clusters (Figure 8a). We found a cluster of GSTs, *GstD3*, *GstD9*, *GstE7*, *GstE9*, *GstE1* and *GstT1,* further interacting with *Ugt36Bc*, *Cyp6a20*, *Cyp4p1* and chaperone *Hsp67Bb*. Notably, *Gclc*, the glutamate-cysteine ligase catalytic subunit, a transcript encoding for the first rate-limiting enzyme of glutathione synthesis, was also on our list. The tetrahydrofolate dehydrogenase/cyclohydrolase *Nmdmc* was also present in our other cluster together with other molecules related to nucleotide metabolism, a glycine dehydrogenase (GLDC) orthologue transcript *CG3999*, a phosphoserine phosphatase transcript *astray* (*aay*) of protein that catalyses the last step in the biosynthesis of serine from carbohydrates, *GART trifunctional enzyme* (*Gart*), which encodes a protein that exhibits phosphoribosylamine-glycine ligase, phosphoribosylformylglycinamidine cyclo-ligase and phosphoribosylglycinamide formyltransferase activities and *Phosphoribosylamidotransferase 2* (*Prat2*), which encodes type-2 glutamine amidotransferase that is essential in the pathway for de novo synthesis of inosine monophosphate (IMP). Transcripts of enzymes tightly linked to serine and threonine metabolism, amino acids fed into the one-carbon metabolism, *Seryl-tRNA synthetase* (*SerRS*) and *Threonyl-tRNA synthetase* (*ThrRS*), were also detected in our cluster. Next, using qRT-PCR analysis, we confirmed that the transcripts for *Nmdmc*, *CG3999*, *aay* and *Gart* were significantly upregulated by *dPerk* overexpression (Figure 9a).

Our analysis also identified a small cluster of genes involved in the processing of single-pass membrane protein with aspartate rich tail 1 (SMDT1) or essential mitochondrial calcium (Ca^2+^) uniporter (MCU) regulator (EMRE) (Figure 8b). EMRE is an essential regulatory subunit of the MCU that mediates Ca^2+^ uptake into mitochondria and is required to bridge the Ca^2+^ sensing proteins mitochondrial calcium uptake 1 (MICU1) and MICU2 with the Ca^2+^ conducting subunit MCU [51,52]. Our cluster identified *Paraplegin* (*Spg7*) and *AFG3 like matrix AAA peptidase subunit 2* (*Afg3l2*), subunits of m-AAA proteases [53,54] and *lethal (2) 37Cc* (*l(2)37Cc*), orthologue of human prohibitin (PHB) and *CG8728*, PMPCA orthologue and *Prohibitin 2* (*Phb2*), which were transcriptionally upregulated, however, their corresponding protein products were not detected. qRT-PCR analysis further confirmed that *dPerk* overexpression resulted in transcriptional upregulation of *Spg7, Afg3l2* and *l(2)37Cc* (Figure 9b). Taken together, our results suggested that dPerk mediates mitochondrial Ca^2+^ levels by restricting the translation of protein products necessary for EMRE processing.

Finally, we identified a network associated with iron-sulphur (Fe-S) clusters (ISCs) (Figure 8c). ISCs are ancient protein cofactors involved in electron transfer reactions that participate in a variety of cellular processes such as enzymatic and redox reactions, respiration, ribosome biogenesis, regulation of gene expression, and DNA-RNA metabolism (reviewed in [55]). In eukaryotes, known Fe-S proteins are located in the mitochondria, cytosol, and nucleus where they perform these diverse functions. Our network includes *Nfs1 cysteine desulphurase* (*Nfs1*) and *bcn92* transcripts, whose proteins together form the L-cysteine desulphurase complex, to which proteins encoded by *Ferredoxins 1* (*Fdx1)* and *2* (*Fdx2)* supply the electrons required for the assembly of the cluster. *Quetzalcoatl* (*Qtzl*) is another transcript whose protein product is predicted to have cysteine desulphurase activity.

We further detected upregulation in the expression of *CG32500*, the protein orthologue of the iron-sulphur cluster scaffold protein NFU1 iron-sulphur cluster scaffold, and *nucleotide binding protein 1* (*Nubp1*), a component of the cytosolic Fe-S cluster assembly (CIA) complex as well as *Maroon-like* (*mal*), whose protein is involved in molybdenum cofactor (Moco) production, a process that is tightly connected to ISC synthesis [56]. Finally, the network included transcripts coding for Ferrochelatase (FeCH), mitochondrial inner membrane Fe-S protein [57], CG6115 an electron transfer flavoprotein regulatory factor 1 orthologue and Cytochrome b5 (Cyt-b5), an electron transport haemoprotein. In conclusion, using STRING MCL clustering, we defined dPerk dependent networks of translationally repressed proteins involved in glutathione and nucleotide metabolism, as well as Ca^2+^ and Fe-S signalling, suggesting that the overexpression of these targets might provide protection against ER stress-related toxicity, as shown with Nmdmc.

## 3. Discussion

PERK activation leads to phosphorylation of eIF2α, a key event in many stress signalling pathways [5]. While this modification leads to global inhibition of protein synthesis, it simultaneously enhances the translation of selected mRNAs, such as that of the transcription factor ATF4 [58], eliciting a gene expression programme designed to confer cellular resistance to stress. We have previously demonstrated that in *pink* and *parkin* mutants, characterised by increased levels of phospho-eIF2α [19], dAtf4 leads to the upregulation of transcripts, whose protein products confer neuroprotection [20], leading us to hypothesise that dPerk target genes that are upregulated at the transcriptional level but fail to be translated consist of failed protective mechanisms.

In this study we aimed to further characterise this subset of upregulated transcripts not met at the level of the proteome in order to define other salvage pathways that bestow cellular protection from ER toxicity.

To assess the relationship between the induction of mRNA and protein levels, we established a dPerk overexpression model that combined microarray analysis and TMT proteomic analysis. We showed that dPerk overexpression leads to phosphorylation of eIF2α and upregulation of the dAtf4-dependent ER stress marker *Nmdmc*, as well as a novel *Drosophila* target *trbl,* thereby confirming that our model reflects two key dPerk signalling characteristics, translational inhibition and canonical ATF4 signalling.

Our findings capture a dynamic transcriptional and translational response to *dPerk* overexpression. While upregulated transcripts suggest an induction of a protective transcriptional programme, our results also show a considerable proportion of downregulated transcripts, as previously reported [59]. These transcripts could be negatively regulated by mRNA turnover or via a specific targeted degradation mechanism, such as IRE1-dependent decay of mRNA (RIDD) [60]. When compared, the differential expression analysis shows a proportion of genes consistently regulated as well as numerous targets differentially regulated, by transcripts and proteins, with the latter indicating that dPerk-dependent translation attenuation creates disparity between transcripts and their protein targets.

We defined two subgroups of upregulated transcripts: genes that were upregulated at both the transcript and protein level (group 1) and genes that were upregulated at the transcript level but not the protein level (group 2). From 27 targets representing group 1, consisting of ER stress, translational, mitochondrial and immune-related molecules, we established *Hsp22* as a dPerk-dependent, dAtf4-dependent target. Hsp22 is a mitochondrial matrix chaperone [61] thought to be involved in the mitochondrial unfolded protein response (UPR^mt^) (reviewed in [62]), suggesting that ER stress may modulate the UPR^mt^ via Hsp22.

Group 2 ClueGO PEA results showed that dPerk overexpression leads to translational repression of mitochondrial proteins. More detailed STRING network analysis identified that previously established nucleotide metabolism was affected, as well as glutathione and Fe-S signalling, all of which are tightly linked to mitochondrial function. A recent study has reported that mitochondrial S-adenosylmethionine (SAM) (mitoSAM) production depends on one-carbon metabolism, and that mitoSAM depletion leads to dysregulation of ISC biosynthesis, linking nucleotide metabolism and ISC synthesis with mitochondrial function [63]. Glutathione metabolism is also related to the methionine cycle, as its product homocysteine can lead to glutathione production via the trans-sulphuration pathway. In accordance with this finding, *ATF4* deficient cells require considerably higher concentrations of cysteine to defend against glutathione depletion [64]. Thus, overexpression of these targets might prove beneficial, as it may help cells adapt to the metabolic consequences of ER stress by modulating mitochondrial function and providing protection against oxidative stress.

Alternatively, the absence of translation itself could represent a mechanism by which a cell can regulate its fate. Our results show that *dPerk* overexpression leads to overexpression of transcripts, though not proteins, of mitochondrial proteases involved in MCU regulatory subunit EMRE processing. The m-AAA proteases have been found to degrade the non-assembled EMRE, and in this manner prevent the assembly of unregulated MCU. Their loss has been determined to result in accumulation of constitutively active MCU-EMRE in neuronal mitochondria, leading to mitochondrial Ca^2+^ overload, mitochondrial permeability transition pore opening, and neuronal death [65]. This finding might help explain the paradox of how activation of UPR and ATF4 might simultaneously lead to cell survival and cell death, as absence of the translational recovery of the UPR, leading to a failure in the production of regulatory proteins might function as an execution signal itself.

Our iRegulon results further support the notion that sustained phosphorylation of eIF2α in dPerk overexpressing flies might not be able to sustain efficient translation of ATF4 targets, as ATF4 was seen as the most strongly associated transcription factor of upregulated transcripts, but not proteins. We demonstrated that *Hsp22* is an dAtf4-dependent target that is also upregulated in proteins; however, *Hsp* mRNAs are efficiently translated [66] due to structural features in their 5′UTRs [67], and translation of Hsp22 was confirmed to be unaffected by rapamycin, a cap-dependent translation inhibitor [68]. This finding suggests that ATF4 leads to upregulation of protective transcripts, however, their translation is dependent on specific translational features of individual transcripts, indicating that the protective response by ATF4 is dependent on the dephosphorylation of eIF2α and thus might be compromised during chronic translational repression. Overexpression of these targets therefore might represent a strategy for combating ER stress related toxicity.

Transcripts like ATF4, which function to relieve stress, are preferentially translated via an uORF-mediated mechanism [58]. Our analysis shows a decrease in uORFs in molecules that are upregulated at the transcript and protein levels (group 1). This suggests that the presence of an uORF is insufficient to predict the resistance to eIF2-dependant translational repression, as previously reported [69,70]. It is known that uORFs can function as both repressors and enhancers of translation (reviewed in [71]). It is therefore reasonable to argue that specific properties of the uORFs are critical for their capacity to regulate translation. However, our analysis did not consider the non-canonical initiation codons (e.g., CUG, UUG, and GUG) for uORF prevalence approximation, which have also been shown to serve as competent sites of translation initiation [72,73,74]. Thus, further work is needed to define the role of group 1 uORFs in translation during ER stress.

Our study has some limitations. First, we used the ubiquitous expression of dPerk, which may mask tissue-specific responses. Second, the data acquired by the proteomics analysis contained fewer molecules than those detected by transcriptomic analysis. This discrepancy might underestimate or overestimate the cellular signatures by the PEA, skewing the interpretation of our results.

A recent study using an astrocytoma cell line reported an increase in the flux of metabolites through one-carbon metabolism [75]. Our study confirms that dPerk regulates one-carbon metabolism genes transcriptionally but indicates that the protein levels of, at least some of these enzymes, are not strongly affected by dPerk upregulation in flies. Nevertheless, it highlights one-carbon metabolism as a major metabolic pathway under the control of dPerk and suggests that its manipulation might have therapeutic potential in diseases associated with PERK activation.

## 4. Materials and Methods

### 4.1. Genetics and Drosophila Strains

Fly stocks and crosses were maintained on standard cornmeal agar media. The crosses were made and maintained at 18 °C during development. For induction of dPerk expression, 1-day-old flies were kept at 29 °C for 15 h, followed by assay execution. Strains used were *da*Gal4, *tub*Gal80ts and *w^1118^* (Bloomington *Drosophila* Stock Center, Bloomington, IN, USA), ATF4 RNAi line (ID: 109014, Vienna Drosophila RNAi Centre, Vienna, Austria). HA-tagged cDNA fragments encoding full-length dPerk (clone ID: LD41715) from the Drosophila Genomics Resource Center (Bloomington, IN, USA) was cloned into pUASTattB vector for PhiC31-mediated site-directed transgenesis. A kinase-dead dPerk mutant was made by mutating lysine 671 to arginine (K671R) using the Quickchange site-directed mutagenesis system (Stratagene, La Jolla, CA, USA). The corresponding transgenic flies were generated at the Cambridge fly facility, Department of Genetics, University of Cambridge (Cambridge, UK). All the experiments on adult flies were performed with males.

### 4.2. Microarray Acquisition and Analysis

The crosses were kept at 18 °C. Following eclosion, adult flies were transferred to 29 °C for 15 h after which the RNA was extracted using the TRIZOL method (4 biological replicates, 5 replicates for each genotype). *dPerk* was driven by *da*GAL4, under control of a temperature sensitive tubGal80 driver. The RNA quality was confirmed using an Agilent 2100 Bioanalyzer (Agilent Technologies, CA, USA). Detailed experimental protocols and raw data were deposited in ArrayExpress under accession E-MTAB-6097. Differential expression was analysed using the Partek Genomics Suite (Partek Inc., St. Louis, MO, USA).

### 4.3. Proteomics Analysis

Protein extracts from whole flies were obtained from 2-day-old male adult flies and heat shocked at 29 °C for 15 h. Extracts were prepared from control (5 biological replicates with the genotype *tub*Gal80, *da*GAL4 > +) and *dPerk* overexpressing flies (5 biological replicates with the genotype *tub*Gal80, *da*GAL4 > UAS-*dPerk*) by grinding flies in radioimmunoprecipitation assay buffer (RIPA) (20 mM Tris pH 7.5, 150 mM NaCl, 1% (*v*/*v*) Nonidet P40, 0.5% (*w*/*v*) sodium deoxycholate, and 1 mM EDTA) supplemented with 1 µg/mL leupeptin, 1 µg/mL antipain, 1 µg/mL chymostatin, 1 µg/mL pepstatin and phosphatase inhibitor cocktail (PhosSTOP, Roche, Basel, Switzerland). The suspensions were cleared by centrifugation at 21,000 *g* and 4 °C for 10 min, and the protein concentrations of the supernatants were measured using the Bradford assay (Bio-Rad, Hercules, CA USA). The cleared lysates were stored at −80 °C until proteomic analysis.

TMT labelling was performed according to the manufacturer’s recommended protocol (https://www.thermofisher.com/order/catalogue/product/90110/, accessed on 26 April 2021). One hundred micrograms of each digested protein sample was labelled individually with each of the 10 TMT tags. After labelling, the samples were combined, cleaned on a Sep-Pak C18 cartridge, dried and dissolved in 20 mM ammonium formate (pH 10). TMT peptide fractionation was performed using an Acquity ethylene-bridged hybrid C18 UPLC column (Waters; 2.1 mm i.d. × 150 mm, particle size of 1.7 µm). Dried fractions were separated using the LC-MS/MS method as detailed below. The fractions were combined into pairs (i.e., the first fraction with the middle fraction) and analysed by LC-MS/MS using a Dionex Ultimate 3000 RSLC nanoUPLC (Thermo Fisher Scientific Inc, Waltham, MA, USA) system and a Lumos Orbitrap mass spectrometer (Thermo Fisher Scientific Inc., Waltham, MA, USA).

For data analysis, the raw data files were processed using Proteome Discoverer v2.1 (Thermo Fisher Scientific, Waltham, MA, USA) and Mascot (Matrix Science, Chicago, IL, USA) v2.6. The data were aligned with the UniProt data from *Pseudomonas aeruginosa* (5584 sequences), which is the common repository of adventitious proteins (cRAP, version 1.0). All comparative analyses were performed with the R statistical language. The R package MSnbase [76] was used for the processing of proteomics data. Briefly, this process entailed the removal of missing values (instances where a protein was identified but not quantified in all channels were rejected from further analysis), log2-transformation of the raw data, and subsequent sample normalisation utilising the ‘diff.median’ method in MSnbase (this translates all samples columns such that they all match the grand median). The differential abundances of the proteins were evaluated using the limma package, and the differences in protein abundances were statistically analysed using Student’s *t*-test with their variances moderated by the empirical Bayes method in limma. The *p*-values were adjusted for multiple testing using the Benjamini Hochberg method [77].

### 4.4. RNA Extraction and Quantitative Real-Time PCR

RNA was extracted using TRIzol (Ambion, Waltham, MA, USA) and quantified by Nanodrop. Quantitative real-time PCR with reverse transcription (qRT-PCR) was performed on a real-time cycler (Applied Biosystems, Foster City, CA, USA, 7500 Fast Real-Time PCR Systems) using the SensiFAST SYBER Lo-ROX One-Step Kit (Bioline, London, UK). *Drosophila* gene specific primers were obtained from QIAGEN (Hilden, Germany): trbl RT^2^ qPCR Primer Assay for Fruit Fly (Cat. no. PPD10848A), Hsp22 RT^2^ qPCR Primer Assay for Fruit Fly (NM_001031943) (Cat. no. PPD66894A-200), Dm_l(2)37Cc_1_SG QuantiTect Primer Assay (NM_001169541, NM_165281, NM_057259) (Cat. no. QT00503615), Dm_CG2658_1_SG QuantiTect Primer Assay (NM_206616, NM_130661) (Cat. no. QT00497231), Dm_CG6512_1_SG QuantiTect Primer Assay (NM_168720, NM_168722) (Cat. no. QT00510608), Dm_CG3999_1_SG QuantiTect Primer Assay (NM_141732) (Cat. no. QT00972349), Dm_Nmdmc_1_SG QuantiTect Primer Assay (NM_057581) (Cat. no. QT00503153), Dm_ade3_1_SG QuantiTect Primer Assay (NM_078773) (Cat. no. QT00931490) and Dm_aay_1_SG QuantiTect Primer Assay (QT00961317) (Cat. no. QT00961317). Gene specific primers for the housekeeping gene rp49 (forward, 5′-TGTCCTTCCAGCTTCAAGATGACCATC-3′; reverse, 5′-CTTGGGCTTGCGCCATTTGTG-3′) were ordered from Sigma-Aldrich (St. Louis, MO, USA).

### 4.5. Protein Extraction and Western Blotting

Protein extracts from whole flies were prepared by grinding flies in lysis buffer (20 mM Tris at pH 7.5, 150 mM NaCl, 1% (*v*/*v*) Nonidet-40, 0.5% (*w*/*v*) sodium deoxycholate, 0.1% (*w/v*) containing phosphatase inhibitor cocktail tablets phosSTOP (Roche, Basel, Switzerland)) and the protease inhibitor cocktail tablets cOmplete ULTRA Tablets, Mini, EASYpack (Roche, Basel, Switzerland) at the manufacturer’s recommended dilution. Following a 10 min ice incubation, the suspensions were centrifuged at 21,000 *g* for 10 min at 4 °C. The protein concentration of the supernatant was measured using the Bradford assay (Bio-Rad, Hemel Hempstead, UK). All the supernatants were mixed in 4x Laemmli loading buffer. For SDS-PAGE, equal concentrations of proteins were run in 12.5% gels and transferred onto a methanol-activated PVDF membrane (Millipore, Watford, UK). The membranes were blocked in TBS/T (0.15 M NaCl and 10 mM Tris-HCl, pH = 7.5) containing either 5% (*w*/*v*) dried nonfat milk or 5% (*w*/*v*) BSA for 1 h at room temperature. The membranes were probed with the primary antibody at 4 °C overnight, followed by incubation with complementary HRP-conjugated secondary antibody. Antibody complexes were visualised by enhanced chemiluminescence (SuperSignal West Dura Extended Duration Substrate, Thermo Fisher Scientific Inc., Waltham, MA, USA). Primary antibodies were used at a dilution 1:1000, unless specified otherwise. Primary antibodies included: Rabbit Anti-α-tubulin (Cell Signalling, 2144), Rabbit Anti-EIF2S1 (phospho S51) (Abcam, ab32157), Rabbit Anti-EIF2S1 (Abcam, ab26197), Rat Anti-HA High Affinity (50 mU/mL, Roche 3F10), together with appropriate secondary antibodies diluted either 1:10,000 or 1:50,000. Stripping of the membrane was performed for 30 min at 37 °C using the Restore Western Blot Stripping Buffer reagent (Thermo Fisher Scientific Inc., Waltham, MA, USA).

### 4.6. Functional Pathway Enrichment and Upstream Analysis

Differential expression transcripts and proteins were obtained based on FC and FDR values of ±1.6 and ≤0.05, respectively, using the R package tidyverse [78]. Pathway enrichment analysis was performed using the Cytoscape v3.8.0 application ClueGo [27]. In brief, a list of differentially expressed targets was submitted for GlueGO functional analysis using *Drosophila melanogaster* [27] as the organism of choice and enriched for 2021 updated Gene Ontology (GO) Cellular Component, GO Biological function, GO Molecular Function, KEGG and Reactome ontologies. Only pathways with a *p*-value ≤ 0.05 were considered. Enrichment and right-sided hypergeometric tests were performed using the default Bonferroni step down pV correction. Grouping was based on the leading group term based on the highest significance and using default 0.4 as the Kappa score. Network analysis and MCL clustering were performed using the online STRING database [49] and the Cytoscape stringApp application. In brief, a list of differentially expressed targets was submitted to the online STRING database. Following mapping, nodes and edges were exported to the stringApp Cytoscape application. Markov cluster (MCL) clustering was performed with the inbuilt stringApp MCL clustering option. The MCL inflation parameter was chosen based on the unique STRING functional enrichment analysis of a given cluster, once the functional specificity was reached. The defined subcluster was reintroduced into the full background transcriptomic network, and first neighbours were investigated, in order to find additional functionally related targets not recognised by the proteomics analysis. Upstream analysis was performed using the Cytoscape application iRegulon [26]. In brief, a list of differentially expressed targets was imported into Cytoscape and queried for the most prominent motifs and transcription factors in the *Drosophila melanogaster* 5 kb upstream and the full transcript putative regulatory region.

### 4.7. uORF Analysis

We used the full list of Drosophila uORFs from a recent study by Zhang and colleagues [69]. Briefly, they queried all *Drosophila* genes from the FlyBase database [79] and identified 7036 genes with at least 1 uORF, according to their definition (starting with AUG and ending with a stop codon UAA/UAG/UGA). Based on this list, we identified genes that contain uORFs in our total set of genes, group 1 and group 2. To assess whether the number of genes sampled in group 1 and group 2 differ from random sampling from the total set of genes, we generated 1000 Monte Carlo simulations of 100 normally distributed integer values based on the mean of the total group. We used a one-sample *t*-test to assess whether the tested number of genes in either group 1 or group 2 is different from the mean of the distribution. We considered the alternative hypothesis to be significant if 5% of the simulations were significant at a *p* value < 0.05. For the analysis of uORF features, the number of uORFs per gene were calculated by counting the number of uORFs in all transcripts of a given gene. The uORF length was calculated by subtracting the uORF sequence end with a uORF sequence start site. The detailed script and verifications of this analysis is deposited on GitHub (https://m1gus.github.io/Perk/, accessed on 26 April 2021).

### 4.8. Statistical Analyses

Statistical analyses were performed using GraphPad Prism v9.0.2 (GraphPad Software, San Diego, CA, USA or R Studio v1.4.1103 (R Studio, Boston, MA, USA). The data presentation and the number of biological replicates per experimental variable (*n*) is indicated in the respective figure. Significance is indicated as * for *p* < 0.05, ** for *p* < 0.01, *** for *p* < 0.001, **** for *p* < 0.0001 and ns for *p* ≥ 0.05.

## Figures and Tables

**Table 1:** List of dPerk-dependent targets that show an upregulation of both transcripts and proteins (group 1). Gene name, brief summary of protein function and transcriptomic and proteomic analysis fold change values (FC) are presented. Abbreviations: ATP, adenosine triphosphate; ER, endoplasmic reticulum; GTP, Guanosine-5’-triphosphate; SLC22, Solute carrier family 22; UPR, Unfolded protein response.

Gene Name	Brief Summary	Transcript FC	Protein FC
ER proteins			
PERK	UPR kinase	16.6	4.99
Sil1	Nucleotide exchange factor required for protein translocation and folding in the endoplasmic reticulum	8.58	1.8
AsnS	Asparagine synthase	4.3	2.3
Ire1	UPR kinase and endoribonuclease	2.09	1.71
**Translation**			
Dgp-1	Translational GTPase predicted to be involved in translational elongation	21.2	2.3
CG2017	Translational GTPase predicted to be involved in translational elongation	2.6	1.69
**Detoxification**			
Cyp6a17	Cytochrome P450 enzyme	117	19.03
Cyp9b2	Cytochrome P450 enzyme	6.96	2.28
Cyp4e3	Cytochrome P450 enzyme	3.22	2.97
Cyp4ad1	Cytochrome P450 enzyme	1.61	1.72
Ugt37A3	UDP-glycosyltransferase	69.4	13.27
Ugt86Dd	UDP-glycosyltransferase	3.48	3.34
GstD2	Glutathione S transferase	7.49	3.68
GstE8	Glutathione S transferase	2.7	2.08
**Mitochondrial proteins**			
Hsp22	Mitochondrial molecular chaperone	29.2	28.25
Pepck2	Mitochondrial and cytoplasmic phosphoenolpyruvate carboxykinase, plays a role in gluconeogenesis	2.63	1.65
CG34423	Mitochondrial ATPase inhibitor	2.4	1.96
**Immunity-related proteins**			
Lectin-galC1	Galactose binding protein involved in the induction of bacterial agglutination and cell-cell adhesion	3.26	9.71
BomBc3	Member of the Bombamin family of small, secreted immune-induced peptides that are induced by Toll signalling and may function to confer resistance to bacterial infection	2.56	3.92
**Other**			
CG11911	Predicted to have serine-type endopeptidase activity and be involved in proteolysis	6.96	9.32
Uro	Urate oxidase, involved in allantoin biosynthetic process	3.73	1.79
Ccp84Ab	Predicted to be a structural constituent of chitin-based larval cuticle	1.92	1.77
CG17752	A member of the SLC22 family, predicted to have transmembrane transporter activity	1.8	1.73
CG7632	Predicted to have hydrolase activity	3.57	1.95
CG43402	Uncharacterized protein	6.77	2.2
CG12868	Uncharacterized protein	2.17	2.36
CG31808	Uncharacterized protein	1.84	2.62

## Data Availability

The code for this is available in the GitHub repository (https://m1gus.github.io/Perk/, last updated on 19 April 2021).

## Supplementary Materials

The following are available online at https://www.mdpi.com/article/10.3390/ijms22094598/s1, Table S1: Full list of transcripts and proteins in dPerk overexpressing flies, Table S2: Identification of transcripts positively regulated in dPerk overexpressing flies (FC ≥ 1.6, FDR ≤ 0.05), Table S3: Identification of transcripts negatively regulated in dPerk overexpressing flies (FC ≥−1.6, FDR ≤ 0.05), Table S4: Identification of proteins positively regulated in dPerk overexpressing flies (FC ≥0.7 (log), FDR ≤ 0.05), Table S5: Identification of proteins negatively regulated in dPerk overexpressing flies (FC ≥−0.7 (log), FDR ≤ 0.05), Table S6: iRegulon Results. Putative upstream regulators of transcripts upregulated in dPerk overexpressing flies. This table is related to Figure 2, Table S7: iRegulon Results: Putative upstream regulators of proteins upregulated in dPerk overexpressing flies. This table is related to Figure 2, Table S8: ClueGO Results: Pathway enrichment analysis of upregulated transcripts in dPerk overexpressing flies. This table is related to Figure 3, Table S9: ClueGO Results: Pathway enrichment analysis of upregulated proteins in dPerk overexpressing flies. This table is related to Figure 3, Table S10: ClueGO Results: Pathway enrichment analysis of downregulated transcripts in dPerk overexpressing flies. This table is related to Figure 3, Table S11: ClueGO Results: Pathway enrichment analysis of downregulated proteins in dPerk overexpressing flies. This table is related to Figure 3, Table S12: List of matched transcripts and proteins in dPerk overexpressing flies, Table S13: Genes with upregulated transcripts and proteins in dPerk overexpressing flies (group 1). This table is related to Table 1, Table S14: dPerk-dependent targets differentially regulated by the transcriptome and proteome. Genes that are upregulated at the transcript but not at protein level were analysed (group 2), Table S15: Genes with downregulated transcripts and proteins in dPerk overexpressing flies (group 3), Table S16: uORF analysis of all matched transcripts and proteins in dPerk overexpressing flies. This table is related to Figure 5, Table S17: uORF analysis of group 1 genes. This table is related to Figure 5, Table S18: uORF analysis of group 2 genes. This table is related to Figure 5, Table S19: ClueGO Cellular Component Results: Pathway enrichment analysis of dPerk-dependent targets differentially regulated by the transcriptome and proteome (group 2). This table is related to Figure 7 (left), Table S20: ClueGO Results: Pathway enrichment analysis of dPerk-dependent targets differentially regulated by the transcriptome and proteome (group 2). This table is related to Figure 7 (left), Table S21: ClueGO Results: Pathway enrichment analysis of dPerk-dependant targets downregulated at the transcript and protein level (group 3). This table is related to Figure 7 (right).
