# Supplementary figures and images for: Combined Transcriptomic and Proteomic Analysis of Perk Toxicity Pathways
Source: Int J Mol Sci. 2021 Apr 27;22(9):4598. doi: 10.3390/ijms22094598 (PMC8124185; doi:10.3390/ijms22094598)

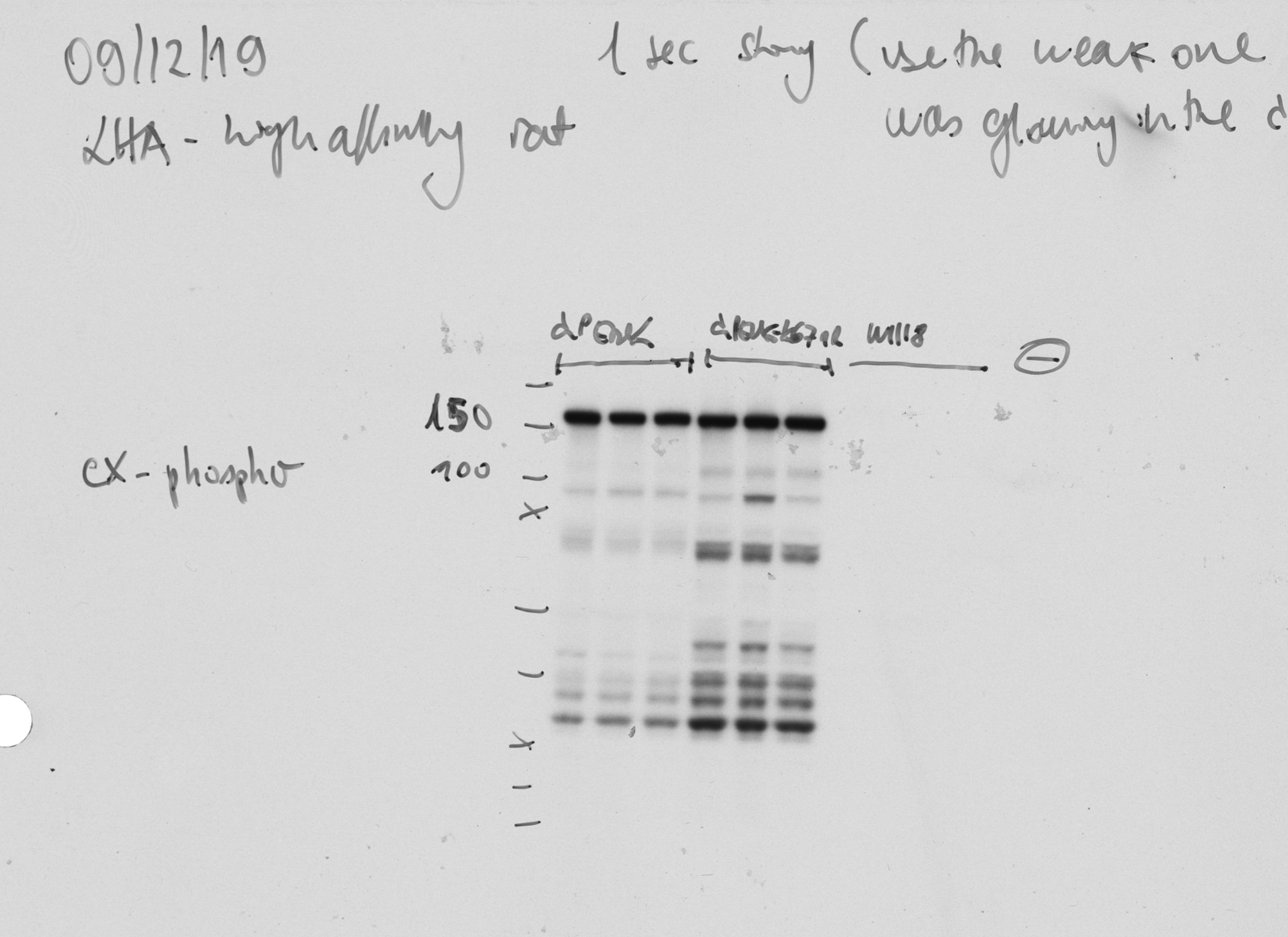

Supplement: Supplementary file 1 [file ijms-22-04598-s001.zip › Figure1d_Original_Data/tubgal80_dPERK_091219_HA.tiff]

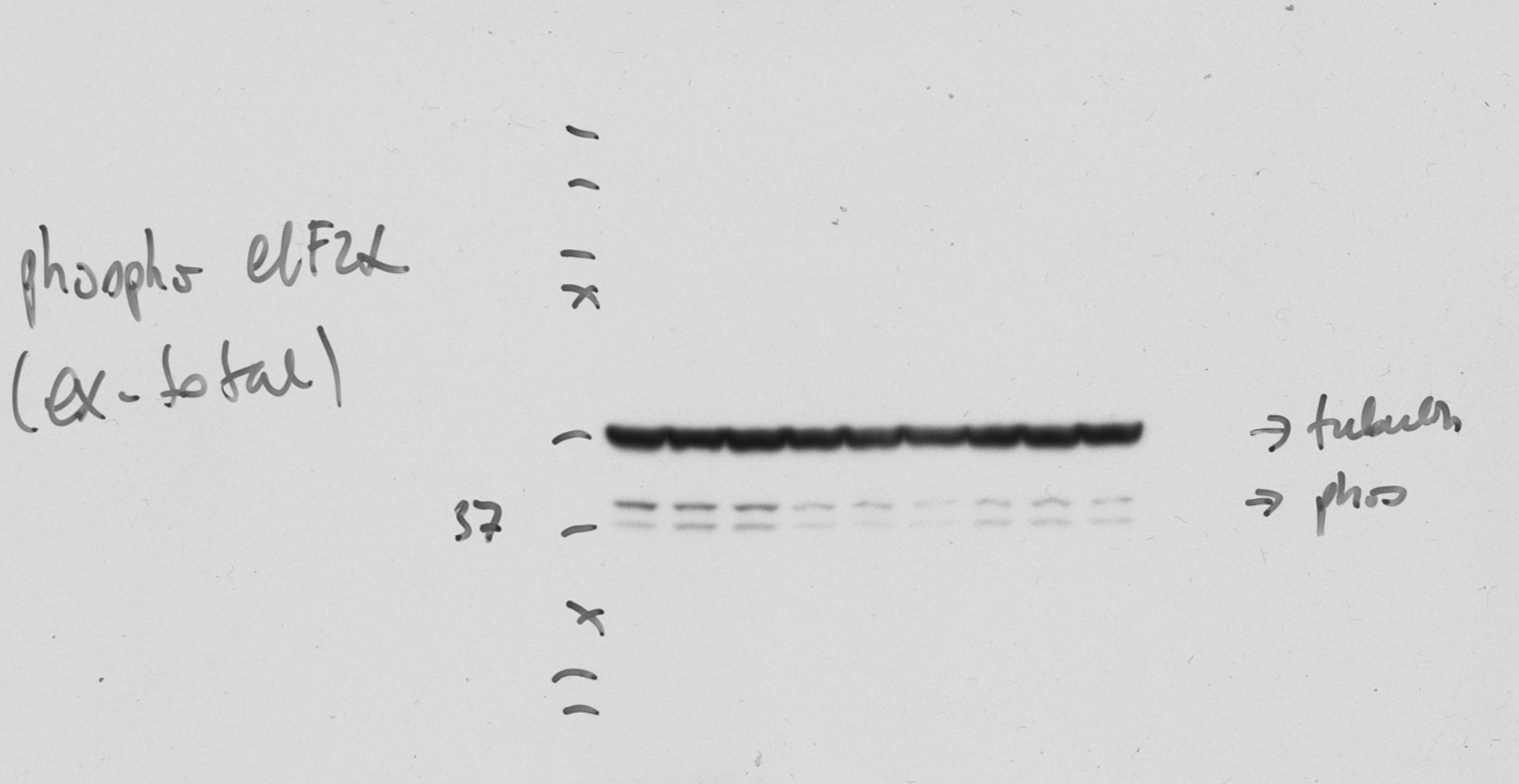

Supplement: Supplementary file 1 [file ijms-22-04598-s001.zip › Figure1d_Original_Data/tubgal80_dPERK_091219_phosphoeIF2a.tiff]

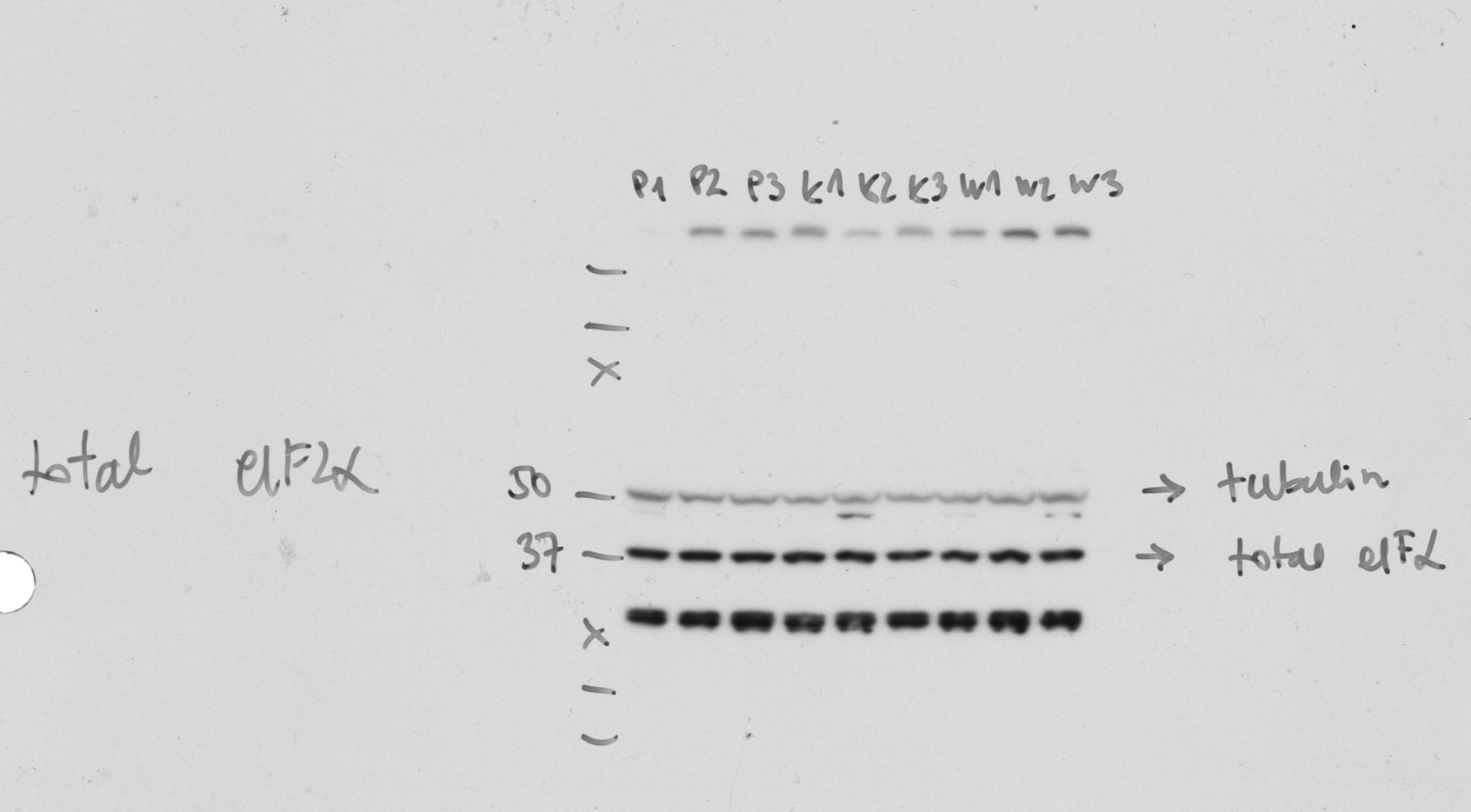

Supplement: Supplementary file 1 [file ijms-22-04598-s001.zip › Figure1d_Original_Data/tubgal80_dPERK_091219_totaleIF2a.tiff]
